# Supplementary material for: Profiles of Polyphenol Intake and Type 2 Diabetes Risk in 60,586 Women Followed for 20 Years: Results from the E3N Cohort Study
Source: Nutrients. 2020 Jun 29;12(7):1934. doi: 10.3390/nu12071934 (PMC7400616; doi:10.3390/nu12071934)
Supplement: Supplementary file 1 [file nutrients-12-01934-s001.pdf]

Table S1: Results of profile regression analysis showing the polyphenols intake profile of each cluster and the resulting logHR associated with each cluster

	Cluster	1	2	3	4	5	6	7	8	9	10	11	12	13	14	15
	N	4228	1925	2983	3661	6298	4664	2827	4546	2280	3299	3118	4810	6272	5432	4243
	Log HR	0.002	0.001	0.001	0.000	0.000	-0.001	-0.001	-0.002	-0.002	-0.002	-0.002	-0.003	-0.003	-0.003	-0.004
	95% CIr	(0.004, 0.000)	(0.003, -0.001)	(0.003, -0.001)	(0.002, -0.001)	Ref	(0.001, -0.003)	(0.001, -0.003)	(0.000. -0.004)	(0.000. -0.005)	(0.000. -0.005)	(0.000. -0.005)	(-0.001, -0.005)	(-0.001, -0.005)	(-0.001, -0.006)	(-0.002, -0.007)
Polyphenol subclasses																
Anthocyanins	Low	0.79	0.65	0.57	0.23	0.56	0.54	0.56	0.35	0.09	0.22	0.06	0.12	0.25	0.04	0.04
	Medium	0.18	0.28	0.33	0.45	0.3	0.34	0.36	0.52	0.23	0.44	0.33	0.35	0.36	0.25	0.22
	High	0.03	0.07	0.1	0.32	0.14	0.11	0.09	0.13	0.68	0.34	0.62	0.53	0.39	0.72	0.74
Dihydrochalcones	Low	0.59	0.49	0.36	0.13	0.4	0.45	0.44	0.62	0.04	0.5	0.25	0.09	0.16	0.26	0.22
	Medium	0.29	0.36	0.4	0.39	0.35	0.37	0.39	0.28	0.15	0.33	0.38	0.28	0.31	0.34	0.34
	High	0.11	0.15	0.24	0.48	0.25	0.18	0.17	0.1	0.81	0.18	0.37	0.63	0.53	0.4	0.44
Dihydroflavonols	Low	0.94	0.53	0.96	0.98	0.6	0.01	0.02	0	0.99	0	0	0.01	0.35	0	0
	Medium	0.06	0.47	0.04	0.02	0.4	0.98	0.97	0	0.01	0	0	0.99	0.65	0	0.01
	High	0	0	0	0	0	0	0.01	1	0	1	1	0	0	1	0.99
Catechins	Low	0.82	0.16	0.68	0.61	0	0.75	0.64	0.66	0.3	0	0.22	0.32	0	0.12	0
	Medium	0.17	0.61	0.3	0.37	0.01	0.24	0.33	0.33	0.61	0.04	0.74	0.63	0.02	0.86	0.13
	High	0.01	0.23	0.03	0.02	0.99	0.01	0.02	0.01	0.09	0.95	0.03	0.05	0.97	0.03	0.86
Proanthocyanidins	Low	0.66	0.54	0.49	0.25	0.5	0.49	0.47	0.56	0.07	0.39	0.11	0.1	0.23	0.08	0.08
	Medium	0.18	0.21	0.26	0.42	0.26	0.3	0.29	0.29	0.31	0.36	0.49	0.38	0.35	0.42	0.39
	High	0.17	0.25	0.25	0.33	0.24	0.21	0.24	0.15	0.62	0.25	0.4	0.52	0.42	0.5	0.53
Flavanones	Low	0.63	0.51	0.36	0.21	0.43	0.47	0.38	0.56	0.08	0.46	0.29	0.1	0.16	0.21	0.18
	Medium	0.25	0.33	0.34	0.44	0.35	0.35	0.36	0.27	0.23	0.33	0.41	0.33	0.32	0.32	0.33
	High	0.12	0.16	0.31	0.36	0.22	0.18	0.26	0.17	0.7	0.21	0.3	0.56	0.52	0.47	0.5
Flavones	Low	0.57	0.54	0.13	0.43	0.48	0.55	0.15	0.47	0.14	0.43	0.41	0.19	0.13	0.18	0.16
	Medium	0.32	0.34	0.27	0.4	0.34	0.34	0.3	0.3	0.3	0.34	0.41	0.36	0.3	0.31	0.33
	High	0.11	0.12	0.6	0.18	0.18	0.11	0.55	0.23	0.56	0.23	0.18	0.45	0.57	0.51	0.52
Flavonols	Low	0.81	0.29	0.59	0.56	0.02	0.72	0.56	0.62	0.28	0.01	0.36	0.28	0.01	0.2	0.01
	Medium	0.16	0.52	0.33	0.35	0.28	0.24	0.36	0.32	0.48	0.26	0.51	0.53	0.15	0.57	0.12
	High	0.03	0.19	0.09	0.09	0.7	0.04	0.08	0.06	0.24	0.73	0.12	0.19	0.84	0.23	0.87

Isoflavonoids	Low	0.41	0.39	0.32	0.39	0.41	0.34	0.27	0.36	0.35	0.36	0.31	0.26	0.29	0.26	0.27
	Medium	0.32	0.31	0.32	0.31	0.32	0.35	0.32	0.32	0.31	0.33	0.36	0.34	0.34	0.34	0.32
	High	0.27	0.3	0.36	0.3	0.27	0.31	0.41	0.32	0.34	0.31	0.34	0.4	0.37	0.4	0.41
Hydroxybenzoic acids	Low	0.91	0.14	0.7	0.69	0	0.81	0.63	0.5	0.33	0	0.21	0.32	0	0.08	0
	Medium	0.09	0.8	0.29	0.31	0.01	0.18	0.36	0.49	0.61	0.02	0.77	0.65	0.02	0.85	0.04
	High	0	0.06	0.01	0.01	0.98	0	0.01	0.01	0.06	0.97	0.02	0.03	0.97	0.08	0.96
Hydroxycinnamic acids	Low	0.32	0.45	0.23	0.34	0.63	0.26	0.15	0.19	0.25	0.55	0.27	0.19	0.49	0.11	0.35
	Medium	0.33	0.32	0.29	0.34	0.24	0.38	0.31	0.38	0.29	0.3	0.44	0.32	0.31	0.34	0.37
	High	0.34	0.23	0.48	0.32	0.12	0.36	0.53	0.43	0.46	0.15	0.29	0.49	0.2	0.55	0.28
Other phenols	Low	0.77	0.57	0.63	0.77	0.58	0.38	0.24	0	0.66	0	0	0.26	0.34	0	0
	Medium	0.18	0.34	0.22	0.18	0.31	0.49	0.45	0.47	0.22	0.38	0.41	0.47	0.4	0.18	0.2
	High	0.05	0.1	0.15	0.05	0.11	0.14	0.31	0.53	0.12	0.62	0.59	0.27	0.26	0.81	0.8
Stilbenes	Low	0.99	0.57	0.98	0.97	0.61	0.02	0.03	0	0.88	0	0	0.01	0.33	0	0
	Medium	0.01	0.43	0.02	0.02	0.39	0.98	0.96	0.01	0.11	0.01	0	0.98	0.66	0	0
	High	0	0	0	0	0	0	0	0.99	0	0.99	1	0.01	0	1	1
Lignans	Low	0.84	0.68	0.26	0.79	0.32	0.72	0.18	0.31	0.39	0.06	0.26	0.33	0.07	0.04	0.01
	Medium	0.12	0.25	0.41	0.16	0.46	0.22	0.43	0.43	0.36	0.41	0.53	0.39	0.34	0.29	0.15
	High	0.03	0.07	0.33	0.04	0.23	0.06	0.39	0.26	0.25	0.53	0.21	0.28	0.59	0.67	0.84
Other polyphenols	Low	0.72	0.69	0.16	0.57	0.66	0.58	0.11	0.29	0.14	0.35	0.29	0.16	0.17	0.03	0.05
	Medium	0.23	0.26	0.31	0.35	0.25	0.34	0.32	0.43	0.35	0.41	0.59	0.39	0.35	0.21	0.26
	High	0.05	0.05	0.53	0.09	0.09	0.07	0.56	0.28	0.51	0.24	0.12	0.46	0.48	0.76	0.69

Clusters generally characterized by women with low, medium and high polyphenols subclasses intakes~~, were~~ are highlighted in red, green and blue, respectively.

Table S2: Adjusted HRs and 95% CIs of T2D according to intakes of total polyphenols and individual subclasses in the E3N cohort.

	Non-cases	Cases	Model 1	Model 2	Model 3
	N (%)	N (%)	HR [95% CI]	HR [95% CI]	HR [95% CI]
**Total polyphenol**	N=57,846	N=2,740			
T1	18992 (32.83)	1001 (36.53)	Reference	Reference	Reference
T2	19104 (33.03)	889 (32.45)	**0.89 [0.81; 0.98]**	**0.90 [0.82; 0.98]**	**0.85 [0.77; 0.94]**
T3	19750 (34.14)	850 (31.02)	**0.86 [0.79; 0.94]**	**0.79 [0.72; 0.86]**	**0.69 [0.61; 0.79]**
**Anthocyanins**	N=57,846	N=2,740			
T1	19063 (32.95)	931 (33.98)	Reference	Reference	Reference
T2	19151 (33.11)	841 (30.69)	**0.84 [0.77; 0.93]**	**0.83 [0.76; 0.91]**	**0.81 [0.74; 0.89]**
T3	19632 (33.94)	968 (35.33)	**0.88 [0.81; 0.97]**	**0.87 [0.80; 0.96]**	**0.82 [0.74; 0.90]**
**Dihydrochalcones**	N=57,846	N=2,740			
T1	19004 (32.85)	989 (36.09)	Reference	Reference	Reference
T2	19119 (33.05)	874 (31.90)	**0.83 [0.76; 0.91]**	**0.84 [0.77; 0.92]**	**0.82 [0.75; 0.90]**
T3	19723 (34.10)	877 (32.01)	**0.76 [0.69; 0.83]**	**0.77 [0.70; 0.84]**	**0.74 [0.67; 0.81]**
**Dihydroflavonols**	N=57,846	N=2,740			
T1	18789 (32.48)	1005 (36.68)	Reference	Reference	Reference
T2	19350 (33.45)	843 (30.77)	**0.82 [0.75; 0.90]**	**0.91 [0.83; 1.00]**	**0.87 [0.79; 0.96]**
T3	19707 (34.07)	892 (32.55)	**0.83 [0.75; 0.90]**	**0.90 [0.82; 0.99]**	**0.76 [0.67; 0.86]**
**Catechins**	N=57,846	N=2,740			
T1	19011 (32.86)	983 (35.88)	Reference	Reference	Reference
T2	19060 (32.95)	933 (34.05)	0.92 [0.84; 1.01]	1.00 [0.91; 1.09]	0.95 [0.87; 1.05]
T3	19775 (34.19)	824 (30.07)	**0.80 [0.73; 0.88]**	0.96 [0.87; 1.05]	0.92 [0.83; 1.01]
**Proanthocyanidins**	N=57,846	N=2,740			
T1	19003 (32.85)	991 (36.17)	Reference	Reference	Reference
T2	19141 (33.09)	852 (31.09)	**0.82 [0.75; 0.90]**	**0.85 [0.77; 0.93]**	**0.81 [0.74; 0.89]**
T3	19702 (34.06)	897 (32.74)	**0.84 [0.77; 0.92]**	**0.87 [0.79; 0.95]**	**0.81 [0.73; 0.89]**
**Flavanones**	N=57,846	N=2,740			
T1	19102 (33.02)	892 (32.55)	Reference	Reference	Reference
T2	19084 (32.99)	909 (33.18)	0.97 [0.88; 1.06]	0.97 [0.88; 1.06]	0.96 [0.87; 1.05]
T3	19660 (33.99)	939 (34.27)	0.96 [0.87; 1.05]	0.96 [0.88; 1.05]	0.95 [0.86; 1.04]
**Flavones**	N=57,846	N=2,740			
T1	19053 (32.94)	941 (34.34)	Reference	Reference	Reference
T2	19109 (33.03)	883 (32.23)	0.93 [0.85; 1.02]	0.98 [0.89; 1.07]	0.94 [0.85; 1.03]
T3	19684 (34.03)	916 (33.43)	0.94 [0.86; 1.03]	0.99 [0.91; 1.09]	0.91 [0.82; 1.02]
**Flavonols**	N=57,846	N=2,740			
T1	19086 (32.99)	908 (33.14)	Reference	Reference	Reference
T2	19060 (32.95)	932 (34.01)	0.99 [0.90; 1.08]	0.93 [0.85; 1.02]	**0.91 [0.83; 1.00]**

T3	19700 (34.06)	900 (32.85)	0.93 [0.85; 1.02]	**0.91 [0.83; 1.00]**	**0.87 [0.79; 0.96]**
**Isoflavonoids**	N=57,846	N=2,740			
T1	19174 (33.15)	915 (33.39)	Reference	Reference	Reference
T2	19010 (32.86)	888 (32.41)	1.00 [0.91; 1.09]	1.02 [0.93; 1.12]	1.01 [0.92; 1.10]
T3	19662 (33.99)	937 (34.20)	**1.09 [0.99; 1.19]**	1.07 [0.97; 1.17]	1.03 [0.94; 1.14]
**Hydroxybenzoic acids**	N=57,846	N=2,740			
T1	19081 (32.99)	913 (33.32)	Reference	Reference	Reference
T2	19013 (32.87)	979 (35.73)	1.02 [0.94; 1.12]	0.98 [0.90; 1.07]	0.95 [0.87; 1.04]
T3	19752 (34.15)	848 (30.95)	**0.88 [0.80; 0.97]**	0.97 [0.88; 1.06]	0.93 [0.84; 1.02]
**Hydroxycinnamic acids**	N=57,846	N=2,740			
T1	19114 (33.04)	880 (32.12)	Reference	Reference	Reference
T2	19070 (32.97)	923 (33.69)	1.05 [0.96; 1.15]	0.96 [0.88; 1.05]	0.98 [0.89; 1.09]
T3	19662 (33.99)	937 (34.20)	**1.11 [1.01; 1.21]**	**0.90 [0.82; 0.99]**	0.97 [0.84; 1.13]
**Other phenols**	N=57,846	N=2,740			
T1	19052 (32.94)	941 (34.34)	Reference	Reference	Reference
T2	19112 (33.04)	882 (32.19)	0.93 [0.85; 1.02]	1.01 [0.92; 1.11]	0.99 [0.90; 1.09]
T3	19682 (34.02)	917 (33.47)	0.95 [0.86; 1.04]	1.01 [0.92; 1.11]	0.94 [0.84; 1.06]
**Stilbenes**	N=57,846	N=2,740			
T1	18978 (32.81)	1016 (37.08)	Reference	Reference	Reference
T2	19169 (33.14)	824 (30.07)	**0.80 [0.73; 0.88]**	**0.88 [0.81; 0.97]**	**0.85 [0.77; 0.93]**
T3	19699 (34.05)	900 (32.85)	**0.83 [0.76; 0.91]**	**0.90 [0.82; 0.99]**	**0.76 [0.67; 0.87]**
**Lignans**	N=57,846	N=2,740			
T1	18952 (32.76)	1041 (37.99)	Reference	Reference	Reference
T2	19131 (33.07)	863 (31.50)	**0.83 [0.76; 0.91]**	**0.91 [0.83; 1.00]**	**0.85 [0.77; 0.93]**
T3	19763 (34.16)	836 (30.51)	**0.79 [0.72; 0.87]**	**0.87 [0.80; 0.96]**	**0.75 [0.67; 0.83]**
**Other polyphenols**	N=57,846	N=2,740			
T1	19008 (32.86)	985 (35.95)	Reference	Reference	Reference
T2	19085 (32.99)	908 (33.14)	**0.91 [0.84; 1.00]**	0.94 [0.86; 1.03]	0.92 [0.84; 1.01]
T3	19753 (34.15)	847 (30.91)	**0.83 [0.76; 0.91]**	**0.86 [0.78; 0.94]**	**0.82 [0.74; 0.91]**

Model 1: adjusted for age (as the time-scale)
Model 2: further adjusted for family history of diabetes, smoking status, physical activity, educational level, hypercholesterolemia, hypertension and BMI
Model 3: further adjusted for total energy, alcohol and coffee
T: tertile

Table S3: Median (interquartile range) intakes of top foods and beverages representatives of the clusters profile of polyphenol intake at higher and lower risk of T2D

	Clusters at higher risk of T2D[1]			Cluster at lower risk of T2D
	cluster_1 (N=4,228)	cluster_2 (N=1,925)	cluster_3 (N=2,983)	cluster_15 (N=4,810)
Polyphenol subclasses (g/day)				
Anthocyanins	28.56 (24.96)	35.40 (26.73)	40.75 (32.01)	84.73 (44.10)
Dihydrochalcones	1.18 (0.97)	1.39 (0.95)	1.71 (1.04)	2.78 (1.31)
Dihydroflavonols	0.00 (0.06)	0.31 (1.12)	0.00 (0.09)	1.54 (1.41)
Catechins	15.27 (15.64)	93.33 (52.60)	23.34 (26.72)	48.06 (39.30)
Proanthocyanidins	101.64 (93.67)	123.13 (139.00)	137.29 (131.67)	258.28 (196.57)
Flavanones	16.26 (18.98)	21.40 (21.95)	32.04 (34.06)	53.30 (36.98)
Flavones	20.35 (10.95)	21.04 (11.17)	41.06 (22.86)	32.87 (17.29)
Flavonols	26.62 (16.58)	47.44 (17.73)	36.29 (20.25)	48.61 (19.27)
Isoflavonoids	0.01 (0.02)	0.01 (0.02)	0.01 (0.02)	0.01 (0.02)
Hydroxybenzoic acids	11.96 (8.34)	38.70 (15.54)	17.76 (11.72)	27.88 (13.76)
Hydroxycinnamic acids	689.66 (674.99)	444.76 (571.30)	896.63 (861.40)	880.76 (687.26)
Other phenolic acids	0.01 (0.06)	0.05 (0.13)	0.02 (0.18)	0.14 (0.23)
Stilbenes	0.07 (0.08)	0.28 (0.73)	0.11 (0.10)	1.17 (0.92)
Lignans	0.28 (0.16)	0.36 (0.17)	0.60 (0.31)	0.52 (0.30)
Other polyphenols	20.13 (11.82)	20.51 (11.39)	45.15 (23.91)	39.90 (22.65)
Food sources (g/day)				
Fresh fruit	136.65 (115.02)	159.18 (123.04)	200.57 (157.69)	351.82 (186.18)
Oleaginous fruits	0.99 (3.94)	1.97 (5.27)	2.96 (7.88)	3.94 (7.09)
Green salad	30.00 (35.71)	34.29 (32.86)	39.29 (38.57)	47.14 (40.00)
Vegetables	114.29 (101.15)	142.86 (115.44)	142.86 (142.86)	171.43 (142.86)
Olive oil	0.00 (0.53)	0.00 (1.05)	0.00 (1.58)	0.79 (2.10)
Green/black tea	0.00 (0.00)	176.35 (110.43)	0.00 (28.57)	14.78 (85.71)
Wine	11.62 (10.21)	11.67 (10.28)	14.83 (11.64)	16.48 (11.31)

[1] Compared to the reference group (cluster 5)

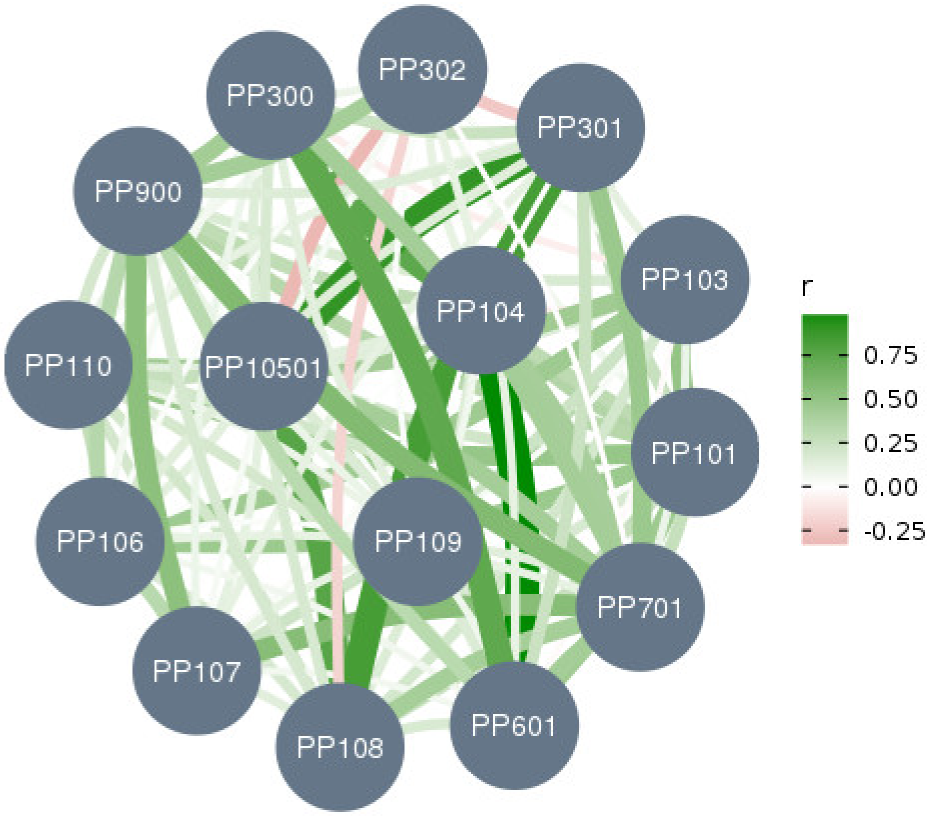

Figure S1: Spearman correlations between intakes of different polyphenol subclasses.

Legend: PP101: Anthocyanins, PP103: Dihydrochalcones, PP104: Dihydroflavonols, PP301: Hydroxybenzoic acids, PP302: Hydroxycinnamic acids, PP300: Other phenolic acids, PP900: Other polyphenols, PP110: Proanthocyanidins, PP106: Flavanones, PP107: Flavones, PP108: Flavonols, PP109: Isoflavonoids, PP601: Stilbenes, PP701: Lignans, PP10501: Catechins
